# Clinics register based HIV prevalence in Jimma zone, Ethiopia: applications of likelihood and Bayesian approaches

**DOI:** 10.1186/s12879-021-06965-0

**Published:** 2022-03-24

**Authors:** Nemso Geda Bedaso, Legesse Kassa Debusho

**Affiliations:** 1 Department of Statistics, College of Natural and Computational Science, Madda Walabu University, Bale Robe, Ethiopia; 2 Department of Statistics, College of Science, Engineering and Technology, University of South Africa, Johannesburg, South Africa

**Keywords:** Bayesian approach, Generalized linear mixed effects model, HIV, Heterogeneity in HIV infection, Maximum likelihood estimation, Odd ratios

## Abstract

**Background:**

The distribution of HIV is not uniform in Ethiopia with some regions recording higher prevalence than others. However, reported regional HIV prevalence estimates mask the heterogeneity of the epidemic within regions. The main purpose of this study was to assess the district differences in HIV prevalence and other factors that affect the prevalence of HIV infection in Jimma zone, Oromia region of Ethiopia. We aimed to identify districts which had higher or lower than zone average HIV prevalence. Such in-depth analysis of HIV data at district level may help to develop effective strategies to reduce the HIV transmission rate.

**Methods:**

Data collected from 8440 patients who were tested for HIV status in government clinics at the 22 Districts between September 2018 to August 2019 in Jimma zone were used for the analyses. A generalized linear mixed effects model with district random effects was applied to assess the factors associated with HIV infection and the best linear unbiased prediction was used to identify districts that had higher or lower HIV infection. Both likelihood and Bayesian methods were considered.

**Results:**

The statistical test on district random effects variance suggested the need for district random effects in all the models. The results from applying both methods on full data show that the odds of HIV infection are significantly associated with covariates considered in this study. Disaggregation of prevalence by gender also highlighted the persistent features of the HIV epidemic in Jimma zone. After controlling for covariates effects, the results from both techniques revealed that there was heterogeneity in HIV infection prevalence among districts within Jimma zone, where some of them had higher and some had lower HIV infection prevalence compared to the zone average HIV infection prevalence.

**Conclusions:**

The study recommends government to give attention to those districts which had higher HIV infection and to conduct further research to improve their intervention strategies. Further, related to those districts which had lower infection, it would be advantageous to identify reasons for their performance and may apply them to overcome HIV infection among residents in those districts which had higher HIV infection. The approach used in this study can also help to assess the effect of interventions introduced by the authorities to control the epidemic and it can easily be extended to assess the regions HIV infection rate relative to the rate at the national level, or zones HIV infection rate relative to the rate at a region level.

**Supplementary Information:**

The online version contains supplementary material available at 10.1186/s12879-021-06965-0.

## Background

Sub-Saharan African region contributes more than two-thirds of the global Human Immunodeficiency Virus (HIV) burden, with 67.4% or 25.6 million HIV cases. The region is hardest hit by HIV in the world, followed by Asia and the Pacific, each with 5.8 and 2.2 million HIV cases, respectively. Of 690,000 of acquired immunodeficiency syndrome (AIDS)-related deaths in 2019, 300,000 and 140,000 occurred in the East and Southern Africa, and West and Central Africa, respectively [1]. An estimated 670,000 people in Ethiopia acquired HIV in 2019, and an estimated 12,000 people died of AIDS-related illness [2]. Of the people living with HIV in 2019, 15,000 people were newly infected cases. The overall national adult HIV prevalence in Ethiopia is estimated at 0.9%, whereas adult women and men prevalence rates are 1.2 and 0.6%, respectively. The figures are much higher in urban areas, about seven times higher than the rural HIV prevalence [3]. The HIV epidemiology of the country is also heterogeneous by geographic areas, where in 2017, Gambella region had the highest prevalence rate (4.8%) followed by the two city administrations Addis Ababa (3.4%) and Dire Dawa (2.5%). However, three fourths of people living with HIV in 2017 were from Amhara (30%) and Oromia (26%) regions, and Addis Ababa (18%) city administration [3]. In addition, it varied by age and socio-economic status.

The estimated HIV prevalence rate in Ethiopia declined from 3.3% in 2000 to 0.9% in 2019, and AIDS-related deaths from 83,000 deaths in 2000 to 15,000 in 2019, thus the country being on the right track to deliver on its commitments. However, the country progress with the first 90% of the Joint United Nations Programme on HIV/AIDS (UNAIDS) 90-90-90 targets by 2020 was not achieved as it was expected [4], where 90-90-90 targets by 2020 means 90% of people living with HIV know their HIV status, 90% of people who know their HIV-positive status are accessing treatment and 90% of people on treatment have suppressed viral loads [5]. The transmission of HIV appeared to continue in the country, particularly among the urban population despite the government persistent efforts to halt the epidemic and HIV remained an issue of public health concern in Ethiopia [6].

The most common modes of HIV transmission are through heterosexual sex, men who have sexual encounters with other women and injecting drug users are also at higher risk of HIV transmission [7]. However, in Ethiopia the most common mode of transmission is through heterosexual sex. Indicators that are related to sexual behaviour risks for HIV infection in the country are age at sexual debut, non-marital and non-cohabiting sexual partnerships, unprotected sexual intercourse, marital status and education level [6]. It has been reported that young females tend to have early sexual debut as compared to males and large per cent of adults who had sexual intercourse with non-marital or non-cohabiting sexual partnerships not using a condom [6]. Studies in Sub-Saharan Africa have shown that HIV prevalence various by age, sex, place of residence and between countries [2, 8].

The HIV distribution in Ethiopia is not uniform [6] with certain regions recording higher prevalence than others. The current national and regional HIV prevalence estimates mask this heterogeneity within the country. To design most effective strategies that help to reduce the HIV transmission rate, it is essential to have a more in-depth analysis of the epidemiological patters and risk factors of HIV in each zone or district in a region. Because targeting high-risk areas with effective control measures yield good results in controlling the pandemic [9]. The main objective of the current study was to assess the factors that affect the prevalence of HIV infection in Jimma zone, Oromia region of Ethiopia. Further, we were interested to assess the district differences in HIV prevalence in Jimma zone and to rank districts by their prevalence rates applying the best linear unbiased prediction (BLUP). Most of the available studies include only public health facilities in Jimma town (e.g. see [10–14]). However, information that shows the comparison of HIV distribution over district to district levels in Jimma zone is very limited. For example, a study conducted by [14] on HIV positive sero-status disclosure and its determinants among people living with HIV/AIDS following ART clinic in Jimma University Specialized Hospital indicates that age, sex, educational status and marital status had significant association with HIV sero-status disclosure. Since study was done only in one hospital, generalization of these findings for entire population of Jimma zone is not possible and comparing the distribution of HIV/AIDS from district to district is difficult.

The rest of the paper is organized as follows. We first describe the data. Next, we specify and outline the likelihood and Bayesian techniques used for model estimation. The results from applying these methods on the study data are presented in Results section. Finally discussion on the results and limitations of the study, and conclusions and pointers for future study are given in Discussion and Conclusion sections, respectively.

## Methods

### Study area and data

The data for this study was obtained from Jimma zone districts’ Public Health center offices. The Jimma zone is in the Oromia regional state and located in the South-Western part of Ethiopia between Latitude 6$^{\circ }$ and 9$^{\circ }$ North and Longitude 34$^{\circ }$ and 38$^{\circ }$ East, and between altitude ranges of 880 to 3340 meters above sea level. The total coverage area of Jimma Zone is 15,568.58 km$^2$ [15].

When patients visit government clinics due to illness, not necessarily HIV related, they get orientation about HIV including means of transmission and advantage of knowing their HIV status by health professionals. Then they are asked if they are willing to do HIV test and those agreed to do the test fill a consent form. The clinics give pre- and post test counselling. All patients aged 15 years and older, who visited the clinics and tested for their HIV status in the 22 districts of Jimma zone during the periods September 2018 to August 2019 were included in this study. The data that were extracted from the patient register include patient HIV status (negative or positive), patient gender, age (15–19, 20–24, 25–49 or $\ge$ 50), marital status (single, married, divorced or widowed), educational level (no education, primary, secondary or superior), condom use (no or yes), religion (protestant, muslim, orthodox and other), occupation (no job, daily worker, farmer, merchant or government employee) and place of residence (rural or urban).

### Ethical consideration

Permission of the study was obtained from Postgraduate research office of Jimma University, College of Natural Sciences. In addition, permission to use the data for this study was obtained from Jimma Zone Ministry of Health Office. The data provided to the authors do not contain any personal identifiers, therefore the anonymity of the patients were assured.

### Statistical methods

#### Generalized linear mixed model

As discussed earlier, HIV prevalence in Ethiopia varies by geographical location. Therefore, this study employed the logistic regression model with random effect to quantify the variation in HIV prevalence that is accounted for by the district variance. Let a binary outcome variable $y_{ij}$ denotes the *j*th patient HIV status in the *i*th district with probability $p_{ij}$, where $y_{ij} = 1$ for tested positive patient and $y_{ij} = 0$ for tested negative patient. A logistic regression model with a random effect for the outcome $y_{ij}$, i.e., a generalized linear mixed effects model (GLMM) is given by

1 $$\begin{aligned} \eta _{ij}= g(\mu _{ij}) = \mathbf{x }_{ij}^{\prime } \,{\varvec{\beta }} + b_i,\quad i = 1, \ldots , m;\quad j=1, \ldots , n_i; \end{aligned}$$

where $g(\cdot )$ is the link function, $\mathbf{x }_{ij} = (1, x_{1ij}, \ldots , x_{pij})$ is vector of *p* explanatory variables or covariates measured on the *j* patient in the *i* district, ${\varvec{\beta }}$ is vector of fixed regression coefficients or parameters, $b_i$ is a random effect varying over districts, $n_i$ is the number of patients in the *i* district and *m* is the number of districts, where for this study $m=22$. The patients living in different districts are likely to vary in their risk of HIV infection and the random effect $b_i$ enables us to include this unknown variation in the model, that is district random effects represent the differences on patients’ HIV test results attributable to the districts but were not captured by any of the covariates $x_{1ij}, \ldots , x_{pij}$. Because $b_i$ is added to all $n_i$ patients for a district, we induce a positive correlation among the $n_i$ responses.

It is assumed that $b_i$ is independently and normally distributed with mean zero and variance $\sigma ^2_b$, in short $b_i \sim N(0, \sigma ^2_b)$. The conditional expectation $\mu _{ij} = p_{ij} = E(y_{ij} = 1 |\mathbf{x }_{ij}, b_i)$ is linked to the linear predictor $\eta _{ij}$ via a link function $g(\cdot )$ and the conditional distribution of $y_{ij}$ belongs to the exponential family. The conditional probability of $y_{ij}$ given the district-specific random effects $b_i$ is given by

$$\begin{aligned} p(y_{ij} =1 | b_i) = p_{ij} = \frac{\exp (\mathbf{x }_{ij}^{\prime }\,{\varvec{\beta }} + b_i)}{1 + \exp (\mathbf{x }_{ij}^{\prime }\,{\varvec{\beta }} + b_i)}. \end{aligned}$$

Assuming that the observations within a district *i* are independent given the random effects $b_i$, the conditional probability of the response vector for the $i^{\text{ th }}$ district $\mathbf{y }_i = (y_{i1}, y_{i2}, \ldots , y_{in_i})^{\prime }$ is given by

$$\begin{aligned} f(\mathbf{y }_i | b_i, {\varvec{\beta }}) = \prod _{j=1}^{n_i}p(y_{ij} = 1 | b_i)^{y_{ij}}p(y_{ij} = 0 | b_i)^{1 - y_{ij}}. \end{aligned}$$

The marginal likelihood function of the *i*th district which is also the likelihood function of ${\varvec{\beta }}$ and a variance of $b_i$, $\sigma ^{2}_b$ is obtained by averaging over the distribution of $b_i$, that is,

2 $$\begin{aligned} L_i({\varvec{\beta }}, \sigma ^2_b ; \mathbf{y }_i) = \int f(\mathbf{y }_i | b_i, {\varvec{\beta }}) f(b_i; \sigma ^{2}_b) d b_i, \end{aligned}$$

where $f(b_i; \sigma ^{2}_b)$ is the probability distribution of $b_i$ with a parameter $\sigma ^2_b$. Recall that $b_i \sim N(0, \sigma ^2_b)$. The total marginal likelihood function is the product of *m* terms in equation (2) and hence

3 $$\begin{aligned} L({\varvec{\beta }}, \sigma ^{2}_b; \mathbf{y }) = \prod _{i=1}^m \int L_i ({\varvec{\beta }}, \sigma ^{2}_b; \mathbf{y }_i), \end{aligned}$$

where $\mathbf{y }=(\mathbf{y }_1^{\prime }, \mathbf{y }_2^{\prime }, \ldots , \mathbf{y }_m^{\prime })^{\prime }$ is the total vector of the responses. Observe that the conditional distribution of $\mathbf{y }_i | b_i$ is not normal, the marginal distribution generally does not have a closed form, hence the integral in Eq. (2) must be approximated in order to run the statistical inference, including estimation of parameters. In this study, we have applied the adaptive Gauss quadrature [16] numerical integration technique to approximate the integral. The combination of Adaptive Gaussian Quadrature for numerical approximation of likelihood function and the Newton–Raphson method for optimization technique produce the most reliable results [17]. A likelihood ratio test was used to test for the associate between individual covariate and HIV prevalence. The asymptotic chi-square mixture distribution [18] test was applied to test $H_0: \sigma ^2_b = 0$ against $H_1: \sigma ^2_b > 0$. The district random effects were estimated using the best linear unbiased predictors (BLUP) procedure after controlling the covriates or fixed effects [19]. The BLUP method is efficient and predicted values obtained using this method are realised values of district random effects [20], therefore it yields the same ranking as true values of random effects [21].

### Bayesian approach for GLMM

The Bayesian approach to GLMMs differs from likelihood methods that it treats all unknown parameters in the model as random variables and have probability distributions called posterior distributions, in contrast to how likelihood methods treat parameters as fixed constants. The likelihood of the model describes the data generating process given the parameters, and the prior usually reflects any previous knowledge about the model parameters. When the prior knowledge is scarce, vague or non-informative priors are assumed so that the posterior distribution is driven by the observed data [22]. The Bayesian aim is to estimate the joint posterior distribution and inference is conducted through the posterior distribution, which combines information from the probability of the data given the parameters, essentially via the likelihood and the prior distributions of the parameters.

Using the Bayes’ theorem, the marginal likelihood can be expressed as [23]

$$\begin{aligned} f(\mathbf{y }_i | {\varvec{\beta }}, \sigma ^{2}_b) = \int \prod _{j=1}^{n_i} f(y_{ij} | {\varvec{\beta }}, b_i)\,f(b_i | \sigma ^{2}_b) d b_i, \end{aligned}$$

where $f(y_{ij} | {\varvec{\beta }}, b_i)$ denotes a probability mass function for HIV status of a patient. As discussed in the previous section, for GLMM this integral usually has no closed form. In such case, it is necessary to resort to other methods to estimate the posterior distribution or, alternatively, to draw samples from it. The classical approach for Bayesian inference is to use the Markov Chain Monte Carlo (MCMC) simulation techniques [24]. However, the MCMC is computationally expensive. Therefore, we have modeled the HIV prevalence using the integrated nested Laplace approximation (INLA) numerical method [25]. For more detailed discussion on the INLA computational approach see, for example, [23, 25, 26].

A Bayesian approach requires the specification of prior distributions for all the random elements of the model. For the GLMM in Eq. (1), it involves choosing priors for the regression coefficients and the hyperparameter of district random effects standard deviation (SD), $\sigma _b$ . In this paper, since empirical information on parameters, ${\varvec{\beta }}$ and $\sigma _b$ or relevant to the study data is not available, non-informative priors were used [27]. Specifically, we have used non-informative priors, i.e. *N*(0, 1000) for the regression coefficients since this is common practice and it allows to compare Bayesian method with maximum likelihood method. Since the choice of priors can have an important impact on posterior distributions of the model parameters and model performance can be sensitive to the choice of the district-specific random effects variance priors [27], we have considered three priors or hyperpriors for $\sigma _b$ precision parameter $\tau _b$, which are based on the inverse gamma distribution [28] and selected the best for the current data using sensitivity analysis. These priors are

(i) $\Gamma (1, 0.0005)$, the default choice of |inla| function in the |R-INLA| package [25];
(ii) $\Gamma (0.001, 0.001)$, the default choice of the BUGS software [29] and it is the most popular choice in Bayesian analysis, and
(iii) $\Gamma (0.5, 0.0164)$, a specification proposed by Fong, Rue and Wakefield [30] and corresponding to random effects $b_i$ with a marginal Cauchy distribution such that $e^{b_i} \in [0.1, 10]$ with probability 0.95.

The fitted models were compared using the Deviance Information Criterion (DIC) [31] and the widely applicable information criteria (WAIC) [32].

For the statistical software program implementation, R codes were written for the likelihood and Bayesian approaches. The GLMMs were fitted using the glmer() function from the lme4 R package [33], whereas the Bayesian analyses were done using the inla() function from the R-INLA package [34]. In addition, ggplot2 and lattice packages of R were used for the graphical outputs. The detailed results are presented in Results section.

## Results

### Exploratory analysis

A total of 8440 patients were tested for HIV infection in government clinic at the 22 Districts between September 2018 to August 2019 in Jimma zone, of them 4228 (50.1%) were men and 4212 (49.9%) were women (see Additional file 1: Table S1). A large per cent (35.1%) of them in the age category 25–49. Over half of them (54.8%) lived in urban areas, only 6% were government employees, only 7.6% of them had above secondary schooling, i.e. superior education level, about 45% of them were muslims, more than a third (38%) were married, and almost half of them (50.4%) did not use condom during sex (Additional file 1: Table S1).

The overall HIV prevalence among tested patients at government clinics in the Jimma zone in the period between September 2018 to August 2019 was 22.1% in women and 24.3% in men (Table 1). In the age group 15–19 years, prevalence was 11.1% in women and 11.7% in men, whereas in the age groups 20–24, 25–49 and 50 years and older the HIV prevalence, respectively were 11.9% in women and 12.2% in men, 14.7% in women and 17.4% in men, and 6.6% in women and 7.2% in men. The HIV prevalence in Jimma zone increased with age, except for age group 50 years and older and was consistently higher among men across all age groups than among women (Table 1). The HIV prevalence was high among married compared to other marital status groups, in those individuals completed primary schooling, daily laborers, among muslims, among urban residents and those who did not use condom during sex (Table 1).

**Table 1:** HIV prevalence by patient characteristics among men and women in Jimma zone, Ethiopia, September 2018 to August 2019

Characteristics	Women ($n = 4212$)	Men ($n= 4228$)	Total ($n = 8440$)
Overall	1867 (22.1%)	2048 (24.3%)	3915 (46.4%)
Age group (years)			
15–19	469 (11.1%)	494 (11.7%)	963 (11.4%)
20–24	500 (11.9%)	514 (12.2%)	1014 (12%)
25–49	621 (14.7%)	735 (17.4%)	1356 (16.1%)
$\ge 50$	277 (6.6%)	305 (7.2%)	582 (6.9%)
Marital status (ref: Single)			
Single	398 (9.4%)	426 (10.1%)	824 (9.8%)
Married	726 (17.2%)	845 (20%)	1571 (18.6%)
Divorced	446 (10.6%)	498 (11.8%)	944 (11.2%)
Widowed	297 (7.1%)	279 (6.6%)	576 (6.8%)
Education level (ref: No education)			
No education	453 (10.8%)	472 (11.2%)	925 (11%)
Primary	873 (20.7%)	1016 (24%)	1889 (22.4%)
Secondary	420 (10%)	428 (10.1%)	848 (10%)
Superior	121 (2.9%)	132 (3.1%)	253 (3%)
Occupation (ref: No job)			
No job	338 (8%)	317 (7.5%)	655 (7.8%)
Daily laborer	646 (15.3%)	803 (19%)	1449 (17.2%)
Farmer	461 (10.9%)	463 (11%)	924 (10.9%)
Government employee	113 (2.7%)	124 (2.9%)	237 (2.8%)
Merchant	309 (7.3%)	341 (8.1%)	650 (7.7%)
Religion (ref: Muslim)			
Muslim	881 (20.9%)	1016 (24%)	1897 (22.5%)
Orthodox	371 (8.8%)	370 (8.8%)	741 (8.8%)
Protestant	615 (14.6%)	662 (15.7%)	1277 (15.1%)
Residence (ref: Rural)			
Rural	837 (19.9%)	815 (19.3%)	1652 (19.6%)
Urban	1030 (24.5%)	1233 (29.2%)	2263 (26.8%)
Condom use (ref: Yes)			
Yes	148 (3.5%)	168 (4%)	316 (3.7%)
No	1719 (40.8%)	1880 (44.5%)	3599 (42.6%)

### Multivariable models

To identify factors associated with HIV infection, a multivariable logistic regression model with district random effects was applied on the data. The covariates were checked for multicollinearity using the variance inflation factor (VIF) before adding them to the model. None of these VIFs (the values are between 1.007 and 1.455) were greater than 5 suggesting the collinearity is not strong to affect the statistical inference in the analysis. To assess effect of a covariate on gender specific prevalence, the analyses were done for each sex separately in addition to analysis done using the combined data.

The estimated variances of the district random effects were ${\hat{\sigma }}^2_b = 0.094, 0.087$ and 0.080 for women, men and full data sets ( Table 2), and for the data sets, the asymptotic chi-square mixture distribution [18] test statistic for testing $H_0: \sigma ^2_b = 0$ against $H_1: \sigma ^2_b > 0$ take the value 19.86, 10.01 and 35.25 with *p*-value $< 0.0001$, 0.0008 and $< 0.0001$ ( Table 2), respectively. The very small *p*-values strongly suggest a rejection of the null hypothesis $H_0: \sigma ^2_b = 0$ that no district-specific random effects should be included in the model. Therefore, these results imply the need for the district (cluster)-random effects in each model fitted to the data, which suggest that individuals with the same characteristics in different districts may have different HIV status in Jimma zone. The tests for fixed effects ( Table 3) show that except occupation across the three data sets and religion in men data set, the other fixed effects or covariates were significantly associated with odds of patients HIV infection because the 95% confidence intervals do not include 1. The results in Table 3 also show that the test on intercept, $\beta _0 = 0$, is significant suggesting that a patient of 50 years of age or older, who was single, no formal education, muslim, had no job, used a condom during sex and lived in a rural area whose district had a random effect equal to zero had a log-odds of HIV infection different from zero.

**Table 2:** District variance estimates, Wald 95% confidence interval and statistical test in women, men and full data

Data	Estimate (${\hat{\sigma }}^2_b$)	SE	95% CI	$\chi ^2$	*p*-value
Women	0.094	0.033	(0.052, 0.219)	19.86	$< 0.0001$
Men	0.087	0.055	(0.034, 0.526)	10.01	0.0008
Full data	0.080	0.032	(0.041, 0.213)	35.25	$<0.0001$

Note that the odds ratios for the individual variables reported in Table 3 are conditional or cluster-specific measures of association. That is, they are interpreted as having an effect conditional on a district random effect being held constant [35]. Therefore, the interpretation of odds ratios are done here for within district comparisons, that is as district adjusted associations between patient characteristics and HIV prevalence.

**Table 3:** Adjusted odds ratios (aOR) and their 95% confidence intervals (CI) for GLMM in equation (1)

	Adjusted odds ratio (95% Credible interval)		
Predictors	Women	Men	Full
Intercept	0.036 (0.022, 0.059)	0.038 (0.023, 0.062)	0.040 (0.028, 0.057)
Age(Ref: $\ge 50$)			
15–19	1.734 (1.190, 2.527)	1.819 (1.271, 2.602)	1.781 (1.374, 2.308)
20–24	0.707 (0.514, 0.971)	0.925 (0.679, 1.260)	0.816 (0.654, 1.017)
25–49	0.908 (0.667, 1.237)	1.118 (0.835, 1.499)	1.017 (0.823, 1.256) 5
Gender (ref: Male)			
Female			0.860 (0.750, 0.987)
Marital status (ref: Single)			
Married	1.674 (1.253, 2.237)	1.514 (1.143, 2.005)	1.592 (1.301, 1.947)
Divorced	1.513 (1.101, 2.080)	1.425 (1.046, 1.942)	1.481 (1.187, 1.849)
Widowed	1.659 (1.157, 2.377)	1.371 (0.958, 1.961)	1.513 (1.174, 1.949)
Education level (ref: No education)			
Primary	1.633 (1.286, 2.074)	1.471 (1.162, 1.864)	1.544 (1.306, 1.826)
Secondary	1.327 (1.007, 1.749)	1.125 (0.854, 1.482)	1.210 (0.996, 1.470)
Superior	1.319 (0.871, 1.998)	1.158 (0.767, 1.748)	1.237 (0.925, 1.655)
Occupation (ref: No job)			
Daily laborer	1.161 (0.874, 1.540)	1.243 (0.936, 1.650)	1.210 (0.991, 1.478)
Farmer	1.080 (0.795, 1.469)	1.446 (1.053, 1.987)	1.249 (1.002, 1.556)
Government employee	1.005 (0.615, 1.642)	1.396 (0.879, 2.217)	1.215 (0.869, 1.698)
Merchant	1.231 (0.879, 1.724)	1.159 (0.831, 1.617)	1.188 (0.938, 1.505)
Religion (ref: Muslim)			
Orthodox	0.894 (0.682, 1.172)	0.870 (0.665, 1.140)	0.880 (0.727, 1.064)
Protestant	0.692 (0.556, 0.861)	0.725 (0.585, 0.897)	0.704 (0.604, 0.819)
Residence (ref: Rural)			
Urban	1.132 (0.931, 1.377)	1.290 (1.061, 1.567)	1.213 (1.057, 1.392)
Condom use (ref: Yes)			
No	74.009 (59.651, 91.823)	67.026 (54.395, 82.590)	70.084 (60.381, 81.347)
*Variance of random effects*			
$\sigma ^2_b (SE)$	0.0936 (0.3059)	0.0869 (0.2948)	0.0795 (0.2820)

Controlling for marital status, education, occupation, religion, place of residence and use of condom during sex, the odds of HIV infection was significantly higher in age group 15–19 years (aOR 1.734, 95% CI 1.190–2.527 for women; aOR 1.819, 95% CI 1.271–2.602 for men; and aOR 1.781, 95% CI 1.374–2.308) in the full data) compared to patients with age 50 years or older but who share the same district average risk, i.e. the same value of the random effect ( Table 3). Whereas the odds of HIV infection for women in age group 20-24 years was significantly lower than women patients with age 50 years or older and lived in the same district (aOR 0.707, 95% CI 0.514–0.971). When comparing patients who lived in the same district and who share identical values on the covariates age, education, occupation, religion, place of residence and use of condom during sex, the odds of HIV infection was 67.4 and 51.4% higher in married women and men compared with single women and men, respectively. Similar trend also observed among divorced and widowed, where the odds of infection among divorced women and men was 51.3 and 42.5% higher than single women and men, respectively; and among widowed it was 65.9 and 37.1% higher than single women and men, respectively. Further, except for widowed men group, the statistical tests on adjusted odds ratio for each of marital status category in the three datasets analyses were statistically significant at 5% level ( Table 3) because the corresponding 95% confidence intervals of the married, divorced and widowed groups for women and full datasets and married and divorced groups for men dataset do not include 1, whereas the confidence interval for widowed men group in mean dataset includes 1.

The odds of HIV infection of women patients who had primary, secondary and superior education levels were 1.633 (95% CI 1.286–2.074), 1.327 (95% CI 1.007–1.749) and 1.319 (95% CI 0.871–1.998) times higher, respectively than the odds of HIV infection of patients with no formal education but who share identical values on the covariates age, marital status, occupation, religion, place of residence and use of condom during sexual intercourse and who also share the same district average risk. Whereas for men patients, the odds 1.471 (95% CI 1.162–1.864), 1.125 (95% CI 0.854–1.482) and 1.158 (95% CI 0.767–1.748) times higher in those who were in primary, secondary and superior education levels compared with those who had no formal education. However, the result was statistically significant only for those in primary education level ( Table 3). For men patients who were farmers, the odds of HIV infection 1.446 (95% CI 1.053–1.987) times significantly higher than those patients who had no job. Compared to muslim patients who share identical values on the covariates age, marital status, education, occupation, place of residence and use of condom during sex and who also shared the same district average risk, the odds of HIV infection was 10.4 (95% CI 0.682–1.172) and 30.8 (95% CI 0.556–0.861) per cent lower in orthodox and protestant women patients, respectively, whereas in men patients it was 13 (95% CI 0.665– 1.40) and 27.5 (95% CI 0.585–0.897) per cents lower in orthodox and protestant patients, respectively. However, the results were statistically significant for protestant patients only (Table 3). Women and men patients lived in urban areas more likely to had HIV infection than those lived in rural areas but who share identical values on the covariates age, marital status, education, occupation, religion and use of condom during sex, and who also share the same district average risk (aOR 1.132, 95% CI 0.931–1.377 for women and aOR ratio 1.290, 95% CI 1.061–1.567 for men) but the result was only significant at 5% level for men patients because its the 95% CI for odds ratio does not include 1. When comparing two patients within the same district and who shared identical values on the remaining six covariates, women and men patients who had not used condom during sex were 74.009 (95% CI 59.651–91.823) and 67.026 (95% CI 54.395–82.590) times more likely to had HIV infection and these results were statistically significant.

### Bayesian approach

**Fig. 1:**
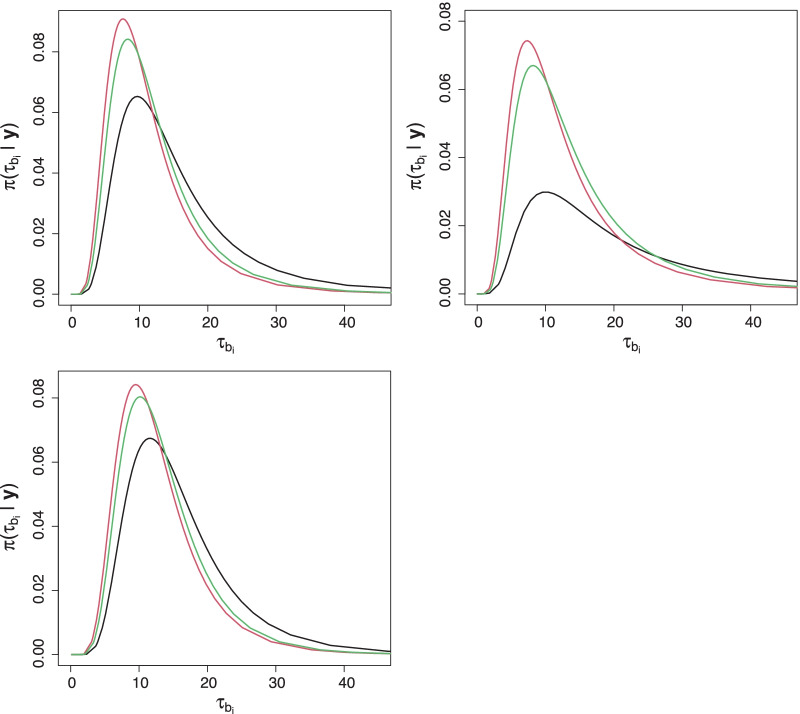
Sensitivity analysis on the priors of precision parameter of fitted models (top left panel for women and tight panel for men and the bottom panel for full data. The black, red and green solid curves are for $\Gamma (1, 0.0005)$, $\Gamma (0.001, 0.001)$ and $\Gamma (0.5, 0.0164)$ priors, respectively

Additional file 1: Tables S2 to S4 report on the results of priors influence on model parameters (posterior means and standard deviations). The three priors of hyperparameter for variance of district-specific random effects yielded similar results for the regression coefficients in models fitted to women, men and full data sets. However, there were slight differences in the point estimates of district random effects variances. Moreover, there were variations in the posterior densities of precision $\tau _{b}$, where $\tau _b = \sigma ^{-1}_b$ across three priors (see Fig. 1). The figure reveals that the Lunn et al. [29] prior was slightly more informative compared to Fong, Rue and Wakefield [30] prior but the inla default prior was the least informative.

**Fig. 2:**
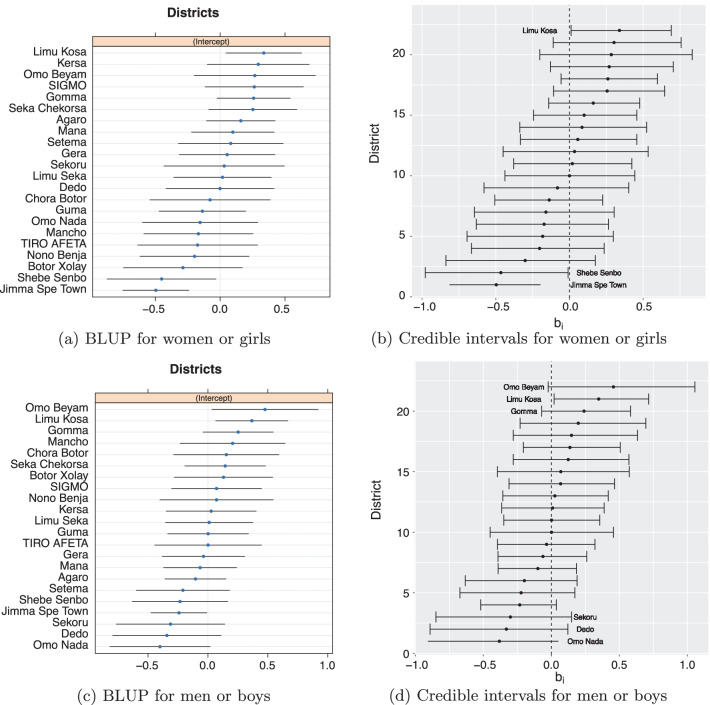
Caterpillar plot for predictors of the random effects of districts with its 95% confidence intervals using BLUP and posterior means (dots) with 95% credible intervals of the random effects of districts in Jimma zone

Table4 displays the DIC and WAIC for the models fitted to the women, men and full data sets using different priors. The DIC and WAIC values of Lunn et al. prior were slightly smaller for models fitted to women and full data sets, however the DIC and WAIC values of Fong, Rue and Wakefield prior were slightly smaller for model fitted to men data than those of the inla default prior and the Lunn et al. prior ( Table 4). These results suggesting that the Lunn et al. prior to be a preferred prior for women and full data sets, whereas the Fong, Rue and Wakefield prior to be a preferred prior for the men data set. Therefore, in what follows, we only report results based on these priors for respective data.

**Table 4:** *SummaryofDICandWAICvaluesfor* different priors across the three data sets

		DIC		WAIC		
Prior	Women	Men	Full	Women	Men	Full
$\Gamma (1, 0.0005)$	2854.88	2960.60	5777.02	2855.79	2961.95	5777.91
$\Gamma (0.001, 0.001)$	2853.57	2953.64	5776.39	2854.57	2954.70	5777.40
$\Gamma (0.5, 0.0164)$	2853.59	2953.27	5776.44	2854.58	2954.41	5777.43

The posterior means of the coefficients (Additional file 1: Tables S2 to S4) are very similar to the Adaptive Gaussian Quadrature or the maximum likelihood estimates ( Table 3), which is in line with the theory that non-informative priors should not have effect on the posterior. Table 5 displays adjusted odds ratios (aOR) and the corresponding 95% credible interval (CI) for covariates. Unlike the posterior means, the credible intervals for Bayesian analysis were different from the likelihood confidence intervals. The credible intervals provide a measure of uncertainty and since for the models fitted in this paper the posterior distributions of regression coefficients are symmetric these intervals are more robust than their likelihood counterpart [23]. Note in Table 5 that the Lunn et al. prior was used for women and full data sets and the Fong, Rue and Wakefield prior for men data set.

**Table 5:** Bayesian analyses results

Coefficients	Adjusted odds ratio (95% Credible interval)		
	Women	Men	Full Data
Intercept	0.035 (0.021, 0.058)	0.037 (0.023, 0.061)	0.039 (0.027, 0.056)
Age (ref: $\ge$50)			
15–19	1.740 (1.195, 2.536)	1.820 (1.273, 2.604)	1.784 (1.376, 2.312)
20–24	0.706 (0.513, 0.969)	0.923 (0.677, 1.257)	0.815 (0.653, 1.017)
25–49	0.908 (0.667, 1.236)	1.119 (0.835, 1.498)	1.017 (0.823, 1.256)
Gender (ref: Male)			
Female			0.860 (0.750, 0.986)
Marital status (ref: Single)			
Married	1.680 (1.258, 2.245)	1.514 (1.143, 2.005)	1.594 (1.303, 1.950)
Divorced	1.518 (1.105, 2.087)	1.424 (1.045, 1.940)	1.483 (1.188, 1.852)
Widowed	1.665 (1.162, 2.387)	1.371 (0.959, 1.962)	1.515 (1.176, 1.953)
Education level (ref: No education)			
Primary	1.637 (1.290, 2.079)	1.474 (1.164, 1.866)	1.546 (1.307, 1.828)
Secondary	1.329 (1.009, 1.753)	1.125 (0.854, 1.482)	1.210 (0.996, 1.470)
Superior	1.320 (0.872, 2.001)	1.157 (0.767, 1.747)	1.238 (0.926, 1.657)
Occupation (ref: No job)			
Daily laborer	1.163 (0.876, 1.542)	1.246 (0.938, 1.652)	1.211 (0.992, 1.479)
Farmer	1.081 (0.795, 1.470)	1.451 (1.057, 1.993)	1.249 (1.002, 1.557)
Government employee	1.006 (0.617, 1.648)	1.402 (0.883, 2.227)	1.215 (0.870, 1.700)
Merchant	1.232 (0.880, 1.726)	1.162 (0.833, 1.620)	1.189 (0.938, 1.506)
Religion (ref: Muslim)			
Orthodox	0.893 (0.681, 1.171)	0.870 (0.664, 1.140)	0.879 (0.727, 1.064)
Protestant	0.691 (0.555, 0.859)	0.724 (0.584, 0.896)	0.703 (0.604, 0.819)
Residence (ref: Rural)			
Urban	1.133 (0.932, 1.378)	1.291 (1.063, 1.569)	1.213 (1.057, 1.392)
Condom use (ref: Yes)			
No	76.470 (61.875, 95.121)	68.848 (56.093, 84.997)	71.307 (61.538, 82.863)
*Random effects*	SD	Median	95% Credible interval
$\sigma _b$ for women	0.0847	0.3164	(0.1815, 0.5147)
$\sigma _b$ for men	0.0918	0.2847	(0.1358, 0.4957)
$\sigma _b$ for full data	0.0690	0.2928	(0.1847, 0.4560)

Considering the credible interval results in the multivariable model with Lunn et al. prior for variance precision, overall the odds of HIV infection among women patients visited district government clinics for various health related problems between September 2018 and August 2019 in Jimma zone was associated with all the covariates. Controlling for other covraiates, the odds of HIV infection among women in age group 15-19 years was 74% (aOR 1.74, 95% CI 1.195–2.536) more than those women with age 50 years or older ( Table 5). However, for those in age groups between 20 and 24, and between 25 and 49, the odds were 29.4 (aOR 0.706, 95% CI 0.513–0.969) and 9.2 (aOR 0.908, 95% CI 0.667 - 1.236) per cents lower than those of adults with age 50 years or older, respectively. Under the Fong, Rue and Wakefield prior, the odds of HIV infection for men in age group 15–19 years was 82% (aOR 1.82, 95% CI 1.273–2.604) higher than those men with age 50 years or older. Similarly, for men patients in age group 25-49 years the odds was 11.9% higher than those men with age 50 years or older. However, in age group 20–24 the odds was 7.7% (aOR 0.923; 95% CI 0.677–1.257) lower compared with the odds of HIV infection in age group 50 years or older. The results revealed that odds of HIV infection among women was 14% (aOR 0.860, 95% CI 0.750–0.986) lower than males (Table 5). Controlling for age, education, occupation, religion, place of residence and use of condom during sexual intercourse of patients, married, divorced and widowed women patients, respectively were 68, 51.8 and 66.5% more likely to had HIV infection compared to unmarried or single women patients (aOR 1.680, 95% CI 1.258–2.245 for married; aOR 1.518, 95% CI 1.665–2.087 for divorced; and aOR 1.665, 95% CI 1.162–2.387 for widowed). Similarly, the odds of HIV infection among married, divorced and widowed men patients were 51.4 (aOR 1.514; 95% CI 1.143–2.005), 42.4 (aOR 1.424, 95% CI 1.045–1.940) and 37.1 (aOR 1.371, 95% CI 0.959–1.962) per cent higher than single men, respectively.

In addition, women with primary, secondary and superior education levels were 63.7, 32.9 and 32.0% more likely to had HIV infection, respectively compared to those women with no formal education (aOR 1.637, 95% CI 1.290–2.079 for primary; aOR 1.329, 95% CI 1.009–1.753; and aOR 1.320, 95% CI 0.872–2.001). However, the increased in odds of HIV infection was statistically nonsignificant for superior education level. Among men patients, there were higher odds of HIV infection in primary (aOR 1.474, 95% CI 1.164–1.866), secondary (aOR 1.125, 95% CI 0.854–1.482) and superior (aOR 1.157, 95% CI 0.767–1.747) education levels relative to men with no formal education but the result was significant only for primary education level. Also, women daily laborers, farmers, government employees and merchants were 16.3, 8.1, 0.6 and 23.2% more at risk of HIV infection, respectively compared to those who were without job, whereas men patients in these categories were 24.6, 45.1 and 16.2 cents more at risk of HIV infection, respectively compared to those who were without job but the increase in odds of HIV infection was statistically significant only in men farmers (aOR 1.451, 95% CI 1.057–1.993). Further, for women the odds of HIV infection among orthodox and protestant were 10.7 and 30.1% lower than muslims (aOR 0.906, 95% CI 0.754, 1.090 for orthodox; and aOR 0.698, 95% CI 0.602, 0.809 for protestant), whereas for men the odds were 13 and 27.6% lower in orthodox and protestant relative to muslims (aOR 0.870, 95% CI 0.664–1.140 for orthodox and aOR 0.724, 95% CI 0.584–0.896 for protestant). For women who were urban residents compared to those who were in rural areas, the odds of HIV infection was 13.3% more (aOR 1.133, 95% CI 0.932–1.378), whereas for men urban residents the odds of HIV infection was 29.1% higher compared to rural residents (aOR 1.291, 95% CI 1.063–1.569). The results also showed that women who had sex without condom were 76.47 times more at risk of having HIV infection compared to those who used condom during sex (aOR 70.611, 95% CI 61.068, 81.872) and the risk was also very high for men who had sex without condom (aOR 68.848, 95% CI 56.093–84.997).

By transforming the quantiles of $\sigma _b$, i.e. by exponentiating median and a 95% credible interval, one can easily interpret the standard deviation on an odds scale. For the results in Table 5, in odds scale median and a 95% credible interval for women are 1.372 and (1.199, 1.673), for men are 1.329 and (1.145, 1.642), respectively. This suggests that one standard deviation in district variation would multiply the odds of HIV infection for women on average by about 1.372, or equally possible divide them by 1.372, and the multiplication also would vary between 1.199 and 1.673 with 0.95 probability. Therefore, the variation in odds of HIV infection among districts was heterogeneous for both women and men in Jimma zone.

## District random effects

Figure 2 shows the caterpillar plots with best linear unbiased prediction (BLUP) values of the districts random effects (left panel) and the posterior means (i.e., the dots in the right panels) and 95% credible intervals of the districts random effects (right panel). The blue dots in the left top and left bottom panels are the conditional modes with error bars. A negative BLUP and posterior mean values for a district associated with a lower HIV prevalence rate compared to the average HIV prevalence rate of Jimma zone while a positive value associated with a higher HIV prevalence rate. Therefore, the top plots both in the left and right panels suggest that Limu Kossa had significantly higher, whereas Jimma special town and Shebe Sembo districts had significantly lower HIV prevalence rate than Jimma zone average HIV prevalence rate among women. Similarly, the bottom left panel shows that Omo Beyam and Limu Kosa districts had significantly higher, whereas Omo Nada and Jimma special town districts had significantly lower HIV prevalence rate, however the right panel shows only Limu Kosa district which had significantly higher HIV prevalence rate compared to the average HIV prevalence rate of Jimma zone among men. Although, Omo Beyam and Omo Nada had large positive and small negative posterior means for men data, respectively their 95% credible intervals in Fig. 2d show that their HIV prevalence rates were not significantly higher or lower than the Jimma zone average, respectively because the zero line crosses the intervals. The negative and positive BLUP values (and posterior means) for district random effects also show heterogeneity of HIV infection among districts in Jimma zone.

## Discussion

This study focused on analysis of HIV prevalence data collected from district clinics register in Jimma zone, Oromia region, Ethiopia using applications of likelihood and Bayesian techniques. The results from applying both techniques on full data show that the odds of HIV infection significantly associated with age, gender, marital status, education level, type of occupation, religion, place of residence and condom use during sex. These results are in agreement with previous studies [36–38]. The two methods of model fitting provide similar results for all components of the fixed effects ${\varvec{\beta }}$ except the posterior mean of farmer where unlike the likelihood result, in the Bayesian method this posterior mean was statistically significant for men and full data sets. Generally, compared to the maximum likelihood method, the Bayesian method yields accurate estimation of variance components [39] but in small samples the results depend on the selected prior distribution for district variance and this variance is also susceptible to bias [28]. Since the number of districts, i.e., clusters is 22 and the number of patients per districts large (ranged from 147 to 1071), there was very small differences among the standard deviations of district random effects for the three precision priors.

In this study, we have found evidence after controlling for other covariates that, in Jimma zone, the odds of HIV infection was lower in women across all age groups compared with men relative to adults patients who were 50 years or older, this result was different from the findings of Amornkul et al. [37, 38, 40], where the findings in these studies show that females are more likely to test positive for HIV. The odds of HIV infection was very high in both gender among adolescents, i.e. in age group 15-19 years, this might be due to the fact that younger age of initial sexual activity is a risk factor for HIV infection among this age group [41]. However, results from this study show that marital status (married, divorced and widowed) was associated with increased odds of HIV infection, this agreed with findings reported in [13, 36, 42, 43] but it differed from Tlou [44] findings. In each of marital status categories, women had a higher odds of HIV infection. The current result might be related to cultural practices like polygamy, which is allowed in some religion, but there is no published report or information on this related to the study area, or it might be due to low rates of condom use by husbands with their wives and may be due to high rates of extramarital sex by men [45]. The study found that patients with a low level of education generally had the highest odds of HIV infection. Although similar trends were found among women and men, women who were in primary, secondary and superior education levels had a higher risk of HIV infection than men in the same categories. However, unlike women, highly educated men were at more risk of HIV infection, this is in agreement with Chen et al. [38] finding, but the current result was not statistically significant.

Further, the study findings show that relative to patients with no job, those men who were farmers, government employees and daily laborers were at more risk of HIV infection than those women in the same categories. These might be because of the relationship between occupation and sexual behaviour that those with higher incomes are more likely to engage in extra relational sexual encounters [46] and daily laborers associated with higher sexual risk behaviours [47]. However, the current result was statistically significant only for men farmers, this might be explained from authors personal observation where in Ethiopia men farmers travels to urban areas either to sell their farm products and purchase seeds, fertilizers and other farm related equipments, and in most part of the country they gather in bars (which are located in urban areas) to have drinks, as these places usually have sex workers, the alcoholic beverages could make them very vulnerable and encourages them to had more multiple sexual partners. Further, the results revealed that non-muslim patients had a lower risk of HIV infection compared to muslim patients, this result different from Chen et al. [38], where in their study they have observed that non-muslims are more likely to test positive. In addition, the study showed that patients residing in urban areas were at more risk of HIV infection than those in rural areas and the risk was higher for men than women and this result supports the Ethiopian Population-based HIV Impact Assessment report of Ethiopian Public Health Institute [6]. The study also reaffirmed condom use during sex is highly protective against HIV infection for both women and men.

The main strengths of this study is that it has managed to illustrate the risk of HIV infection between women and men after controlling for possible confounders. The disaggregation of HIV prevalence by gender highlights the persistent features of the epidemic in Jimma zone, for example men across all age groups had higher prevalence compared with women in the same age group and the HIV prevalence or odds of HIV infection among married, divorced and widowed women patients higher than men in the same marital status. In addition, the best linear unbiased predictors for district random effects helped to identify the districts that had significant higher or lower HIV prevalence rate than the Jimma zone average HIV prevalence rate. The district random effects represent the differences on patient’s HIV test result attributable to the districts, but were not captured by any of the covariates. These may include whether the local government authorities introduced educational programmes to promote use of condom during sex and encouraging residents to know their HIV status or not. This analysis permits, for example, those districts which had higher than the Jimma zone average HIV prevalence rate to be targeted for further research in order to improve their intervention strategies. Despite these strengths, the study had some limitations. HIV infection prevalence associated with sexual behaviour characteristics such as age at first sex, condom use at first sex and number of sex partners, frequency of HIV testing, HIV knowledge and clinical characteristics, e.g., sexually transmitted infections [37, 38]. However, these information were not introduced in the analysis because the clinic patient register data used in this study do not contain them. Furthermore, in this paper, our analysis of risk factors of HIV infection in Jimma zone was done using clinic register data, therefore the results should be interpreted with caution. The findings are limited to this area and not necessarily generalizable to the Oromia region or the country.

## Conclusion

In this paper, we assessed the differences in HIV prevalence among districts in Jimma zone and ranked them by their rate of HIV prevalence, and also assessed the effects of patients related variables on HIV infection prevalence applying the likelihood and the Bayesian techniques using data collected from patients register at district clinics. The results from applying both techniques show that the odds of HIV infection significantly associated with most of patients related variables. Disaggregation of prevalence by gender highlights the persistent features of the HIV epidemic in Jimma zone, where except for patients who were widowed, unemployed and rural residents, men across the other characteristics had higher prevalence compared with women. In addition to identifying the significant risk factors associated with the prevalence of HIV infection, the generalized linear mixed effects model settings allowed us to compare district HIV infection prevalence rate with the average prevalence of Jimma zone. After controlling for patients variables or characteristics effects, the results from both techniques revealed that there was heterogeneity in HIV infection prevalence among districts within Jimma zone, where some of them had higher and some had lower prevalence rate than the average prevalence rate of Jimma zone.

The study recommends federal or regional authorities to give attention to those districts which had higher HIV prevalence and to conduct further research in order to improve their intervention strategies. Further, related to those districts which had lower HIV prevalence, it would be advantageous to identify reasons for their performance and may apply them to overcome HIV infection among residents in those which had higher prevalence rate. The approach used in this study can also help to assess the effect of interventions introduced by the authorities to control the epidemic and it can easily be extended to assess the region HIV prevalence rate relative to the rate at national level or zones HIV prevalence rate relative to the rate at a region level.

## Supplementary Information

**Additional file 1: Table S1.** Distribution of patient HIV prevalence by their characteristics among men and women inJimma zone, Ethiopia, September 2018 to August 2019. **Table S2.** Parameter estimates of the GLMM in equation (1) for women HIV prevalence data using different choices of priors for district variance. **Table S3.** Parameter estimates of the GLMM in equation (1) for men HIV prevalence data using different choices of priors for district variance. **Table S4.** Parameter estimates of the GLMM in equation (1) for full HIV prevalence data using different choices of priors for district variance.

## Data Availability

The data that support the findings of this study are available from Jimma Zone Ministry of Health Office but restrictions apply to the availability of these data, which were used under license for the current study, and so are not publicly available. Data are however available from the authors upon reasonable request and with permission of Jimma Zone Ministry of Health Office.

## Abbreviations

AIDS: Acquired immuno deficiency syndrome
aOR: Adjusted odds ratio
ARV: Antiretroviral
BLUP: Best linear unbiased prediction
DIC: Deviance Information Criterion
GLMM: Generalized linear mixed model
HIV: Human Immunodeficiency Virus
INLA: Integrated nested Laplace approximation
MCMC: Markov Chain Monte Carlo
OR: Odds ratios
VIF: variance inflation factor
WAIC: Widely applicable information criteria.
