# Supplementary material for: Clinics register based HIV prevalence in Jimma zone, Ethiopia: applications of likelihood and Bayesian approaches
Source: BMC Infect Dis. 2022 Mar 24;22:281. doi: 10.1186/s12879-021-06965-0 (PMC8944036; doi:10.1186/s12879-021-06965-0)
Supplement: Supplementary file 1 — Additional file 1: Table S1. Distribution of patient HIV prevalence by their characteristics among men and women inJimma zone, Ethiopia, September 2018 to August 2019. Table S2. Parameter estimates of the GLMM in equation (1) for women HIV prevalence data using different choices of priors for district variance. Table S3. Parameter estimates of the GLMM in equation (1) for men HIV prevalence data using different choices of priors for district variance. Table S4. Parameter estimates of the GLMM in equation (1) for full HIV prevalence data using different choices of priors for district variance. [file 12879_2021_6965_MOESM1_ESM.pdf]

Clinics Register based HIV Prevalence in Jimma Zone, Ethiopia: Applications of Likelihood and Bayesian Approaches: Additional Data

Nemso Geda Bedaso and Legesse Kassa Debusho*
* Corresponding author: debuslk@unisa.ac.za

Descriptive analysis and χ^2 test of association

Descriptive statistics with χ^2 test of association between patients' characteristic and HIV prevalence are displayed in Table A1. The χ^2 test results show that there was significant association between each of the patients' characteristic and HIV prevalence in the Jimma zone.

Table A1: Distribution of patient HIV prevalence by their characteristics among men and women in Jimma zone, Ethiopia, September 2018 to August 2019.

Variables	HIV status: n (%) Negative	Positive	Distribution of patients
Gender			
Female	2345 (27.8)	1867 (22.1)	4212 (49.9)
Male	2180 (25.8)	2048 (24.3)	4228 (50.1)
Age			< 0.0001
15-19	697 (8.3)	963 (11.4)	1660 (18.6)
20-24	1481 (17.5)	1014 (12.0)	2495 (28.0)
25-49	1607 (19.0)	1356 (16.1)	2963 (33.2)
>=50	740 (8.8)	582 (6.9)	1322 (14.5)
Marital status			< 0.0001
Single	1010 (12.0)	824 (9.8)	1834 (21.8)
Married	1635 (19.4)	1571 (18.6)	3206 (38.0)
Divorced	1206 (14.3)	944 (11.2)	2150 (25.5)
Widowed	674 (8.0)	576 (6.8)	1250 (14.8)
Education level			< 0.0001
No education	1436 (16.9)	925 (11.0)	2355 (27.9)
Primary	1655 (19.6)	1889 (22.4)	3544 (42.0)
Secondary	1053 (12.5)	848 (10.0)	1901 (22.5)
Superior	387 (4.6)	253 (3.0)	640 (7.6)
Occupation			0.0237
No job	794 (9.4)	655 (7.8)	1449 (17.2)
Daily worker	1649 (19.5)	1449 (17.2)	3098 (36.7)
Farmer	1008 (11.9)	924 (10.9)	1932 (22.8)
Government employee	272 (3.2)	237 (2.8)	509 (6.0)
Merchant	802 (9.5)	650 (7.7)	1452 (17.2)
Religion			< 0.0001
Muslim	1901 (22.5)	1897 (22.5)	3798 (45.0)
Protestant	1863 (22.1)	1277 (15.1)	3140 (37.2)
Orthodox & others	761 (9.0)	741 (8.8)	1502 (17.8)
Residence			< 0.0001
Rural	2164 (25.6)	1652 (19.6)	3816 (45.2)
Urban	2361 (28.0)	2263 (26.8)	4624 (54.8)
Condom use			< 0.0001
Yes	3867 (45.8)	316 (3.7)	4183 (49.5)
No	658 (7.8)	3599 (42.6)	4257 (50.4)

Results of Bayesian analyses

Table A2: Parameter estimates of the GLMM in equation (1) for women HIV prevalence data using different choices of priors for district variance.

	Priors					
	$\Gamma(1, 0.0005)$		$\Gamma(0.001, 0.001)$		$\Gamma(0.5, 0.0164)$	
Coefficients	Mean	SD	Mean	SD	Mean	SD
Intercept	-3.328	0.250	-3.345	0.253	-3.340	0.252
Age (ref: >=50)						
$15-19$	0.547	0.192	0.554	0.192	0.552	0.192
$20-24$	-0.349	0.162	-0.349	0.162	-0.349	0.162
$25-49$	-0.099	0.157	-0.096	0.157	-0.097	0.157
Marital status (ref: Single)						
Married	0.512	0.147	0.519	0.148	0.517	0.147
Divorced	0.411	0.162	0.418	0.162	0.416	0.162
Widowed	0.503	0.183	0.510	0.183	0.508	0.183
Education level (ref: No education) Primary	0.493	0.122	0.493	0.122	0.493	0.122
Secondary	0.285	0.140	0.285	0.141	0.285	0.141
Superior	0.276	0.211	0.278	0.212	0.277	0.212
Occupation (ref: No job)						
Daily laborer	0.151	0.144	0.151	0.144	0.151	0.144
Farmer	0.081	0.156	0.078	0.157	0.079	0.157
Government employee	0.010	0.250	0.006	0.250	0.007	0.250
Merchant	0.210	0.171	0.208	0.172	0.209	0.172
Religion (ref: Muslim)						
Orthodox	-0.110	0.138	-0.113	0.138	-0.112	0.138
Protestant	-0.368	0.111	-0.370	0.111	-0.369	0.111
Residence (ref: Rural)						
Urban	0.125	0.100	0.125	0.100	0.125	0.100
Condom use (ref: Yes)						
No	4.325	0.110	4.337	0.110	4.334	0.109
Random effect						
σ_b	0.278	0.080	0.325	0.085	0.311	0.080

Table A3: Parameter estimates of the GLMM in equation (1) for men HIV prevalence data using different choices of priors for district variance.

	Priors					
	$\Gamma(1, 0.0005)$		$\Gamma(0.001, 0.001)$		$\Gamma(0.5, 0.0164)$	
Coefficients	Mean	SD	Mean	SD	Mean	SD
Intercept	-3.259	0.245	-3.288	0.252	-3.285	0.250
Age (ref: >=50)						
$15-19$	0.584	0.182	0.600	0.183	0.599	0.182
$20-24$	-0.093	0.157	-0.079	0.158	-0.080	0.158
$25-49$	0.109	0.149	0.113	0.149	0.113	0.149
Marital status (ref: Single) Married	0.403	0.143	0.416	0.143	0.415	0.143
Divorced	0.337	0.158	0.355	0.158	0.353	0.158
Widowed	0.305	0.182	0.316	0.182	0.315	0.182
Education level (ref: No education) Primary	0.386	0.120	0.388	0.120	0.388	0.120
Secondary	0.118	0.140	0.118	0.140	0.118	0.140
Superior	0.140	0.209	0.146	0.210	0.146	0.210
Occupation (ref: No job)						
Daily laborer	0.227	0.144	0.219	0.144	0.220	0.144
Farmer	0.385	0.161	0.371	0.162	0.373	0.162
Government employee	0.361	0.236	0.336	0.236	0.338	0.236
Merchant	0.160	0.169	0.149	0.169	0.150	0.169
Religion (ref: Muslim)						
Orthodox	-0.139	0.137	-0.140	0.137	-0.140	0.137
Protestant	-0.324	0.109	-0.323	0.109	-0.323	0.109
Residence (ref: Rural)						
Urban	0.256	0.099	0.256	0.099	0.256	0.099
Condom use (ref: Yes)						
No	4.205	0.107	4.235	0.106	4.232	0.106
Random effect						
σ_b	0.182	0.120	0.307	0.100	0.293	0.092

Table A4: Parameter estimates of the GLMM in equation (1) for full HIV prevalence data using different choices of priors for district variance.

	Priors					
	$\Gamma(1, 0.0005)$		$\Gamma(0.001, 0.001)$		$\Gamma(0.5, 0.0164)$	
	Mean	SD	Mean	SD	Mean	SD
Intercept	-3.231	0.185	-3.238	0.187	-3.236	0.186
Age (ref: >=50)						
$15-19$	0.576	0.132	0.579	0.132	0.578	0.132
$20-24$	-0.205	0.113	-0.204	0.113	-0.204	0.113
$25-49$	0.016	0.108	0.017	0.108	0.017	0.108
Gender (ref: Male)						
Female	-0.150	0.070	-0.151	0.070	-0.151	0.070
Marital status (ref: Single)						
Married	0.464	0.103	0.466	0.103	0.466	0.103
Divorced	0.391	0.113	0.394	0.113	0.394	0.113
Widowed	0.413	0.129	0.416	0.129	0.415	0.129
Education level (ref: No education)						
Primary	0.435	0.085	0.435	0.085	0.435	0.085
Secondary	0.191	0.099	0.191	0.099	0.191	0.099
Superior	0.212	0.148	0.213	0.148	0.213	0.148
Occupation (ref: No job)						
Daily laborer	0.192	0.102	0.192	0.102	0.192	0.102
Farmer	0.224	0.112	0.222	0.112	0.223	0.112
Government employee	0.196	0.171	0.195	0.171	0.195	0.171
Merchant	0.174	0.120	0.173	0.120	0.173	0.120
Religion (ref: Muslim)						
Orthodox	-0.128	0.097	-0.129	0.097	-0.129	0.097
Protestant	-0.352	0.078	-0.352	0.078	-0.352	0.078
Residence (ref: Rural)						
Urban	0.193	0.070	0.193	0.070	0.193	0.070
Condom use (ref: Yes)						
No	4.261	0.076	4.267	0.076	4.265	0.076
Random effect						
σ_b	0.269	0.063	0.300	0.069	0.291	0.066
